# Supplementary material for: Using Ambulatory Care Sensitive Conditions to Assess Primary Health Care Performance during Disasters: A Systematic Review
Source: Int J Environ Res Public Health. 2022 Jul 27;19(15):9193. doi: 10.3390/ijerph19159193 (PMC9367847; doi:10.3390/ijerph19159193)
Supplement: Supplementary file 1 [file ijerph-19-09193-s001.zip › File S1.pdf]

File S1*:* Search Queries for PubMed, Web Of Science and Scopus

PubMed	("ambulatory care sensitive condition*"[Title/Abstract] OR "ambulatory care-sensitive condition*"[Title/Abstract] OR "ACSC*"[Title/Abstract] OR "avoidable hospitalization*"[Title/Abstract] OR "avoidable hospitalisation*"[Title/Abstract] OR "avoidable hospital admission*"[Title/Abstract] OR "preventable hospitalization*"[Title/Abstract] OR "preventable hospitalisation*"[Title/Abstract] OR "preventable health condition*"[Title/Abstract] OR "preventable hospital admission*"[Title/Abstract] OR "preventable condition*"[Title/Abstract] OR "avoidable emergency admission*"[Title/Abstract] OR "preventable emergency admission*"[Title/Abstract] OR "avoidable health conditions*"[Title/Abstract]) AND ((disaster[MeSH Terms]) OR ("disaster*"[Title/Abstract] OR "emergency"[Title/Abstract] OR "emergencies"[Title/Abstract] OR "crisis"[Title/Abstract] OR "crises"[Title/Abstract] OR "catastrophe"[Title/Abstract] OR "covid*"[Title/Abstract] OR "calamity*"[Title/Abstract] OR "flood*"[Title/Abstract] OR "tsunami"[Title/Abstract] OR "earthquake*"[Title/Abstract] OR "storm*"[Title/Abstract] OR "pandemic*"[Title/Abstract] OR "epidemic*"[Title/Abstract] OR "cyclone*"[Title/Abstract] OR "heatwave*"[Title/Abstract] OR "heat wave*"[Title/Abstract] OR "drought*"[Title/Abstract]))
Web Of Science	("ambulatory care sensitive condition*" OR "ambulatory care-sensitive condition*" OR "ACSC*" OR "avoidable hospitalization*" OR "avoidable hospitalisation*" OR "avoidable hospital admission*" OR "preventable hospitalization*" OR "preventable hospitalisation*" OR "preventable health condition*" OR "preventable hospital admission*" OR "preventable condition*" OR "avoidable emergency admission*" OR "preventable emergency admission*" OR "avoidable health conditions*") AND ("disaster*" OR "emergency" OR "emergencies" OR "crisis" OR "crises" OR "catastrophe" OR "covid*" OR "calamity*" OR "flood*" OR "tsunami" OR "earthquake*" OR "storm*" O R "pandemic*" OR "epidemic*" OR "cyclone*" OR "heatwave*" OR "heat wave*" OR "drought*")
SCOPUS	(TITLE-ABS-KEY ("ambulatory care sensitive condition*" OR "ambulatory care-sensitive condition*" OR "ACSC*" OR "avoidable hospitalization*" OR "avoidable hospitalisation*" OR "avoidable hospital admission*" OR "preventable hospitalization*" OR "preventable hospitalisation*" OR "preventable health condition*" OR "preventable hospital admission*" OR "preventable condition*" OR "avoidable emergency admission*" OR "preventable emergency admission*" OR "avoidable health conditions*") AND TITLE-ABS-KEY ("disaster*" OR "emergency" OR "emergencies" OR "crisis" OR "crises" OR "catastrophe" O R "covid*" OR "calamity*" OR "flood*" OR "tsunami" OR "earthquake*" OR "storm*" OR "pandemic*" OR "epidemic*" OR "cyclone*" OR "heatwave*" OR "heat wave*" OR "drought*"))
