# Supplementary material for: Using Ambulatory Care Sensitive Conditions to Assess Primary Health Care Performance during Disasters: A Systematic Review
Source: Int J Environ Res Public Health. 2022 Jul 27;19(15):9193. doi: 10.3390/ijerph19159193 (PMC9367847; doi:10.3390/ijerph19159193)
Supplement: Supplementary file 1 [file ijerph-19-09193-s001.zip › File S3.pdf]

Author	Year	Country	Disaster Type	Data sources/population	Objectives	Results	Limitations
**Sasabuchi Y, et al [18]**	2018	Japan	Earthquake	N=7921 admissions for vaccine-preventable ACSCs<br>N= 18763 for acute ACSCs<br>N= 85436 for chronic ACSCs	To investigate the impact of the 2016 Kumamoto earthquakes on ACSCs, using an administrative database from Kumamoto prefecture	-In the first 7 days after the earthquakes all ACSCs in 2016 increased and then decreased, while number of ACSC admissions did not change in 2013-2015.<br>-Difference-in-differences analysis within 7 days showed that admissions increased for vaccine-preventable, acute and chronic ACSCs.	-No information related to degree of damage of infrastructures<br>-Differences between the population included in the present study and the general population<br>-Validity of ACSC in Japanese clinical setting needs to be further evaluated<br>-Difference in admission policy between hospitals<br>-ACSC are not a direct measure of the impaired of access to primary care.
**Huang, Y.-T., et al. [19]**	2009	Taiwan	SARS outbreak	N= 120.330 hospitalizations	To explore the effect of the SARS outbreak on hospitalization from chronic ACSCs after the SARS outbreak<br><br>01-03/2003 → pre-SARS period<br>04-06/2003 → SARS<br>07-09/2003 → post-SARS-1<br>10-12/2003→ post-SARS-2	-ACSC hospitalizations were reduced during the SARS period<br>-In the post-1 phase hospitalizations were still reduced with exception of diabetes and arterial hypertension whose hospitalization rates increased after SARS.<br>-Actual hosp. rates were lower than predicted (except for diabetes and arterial hypertension)	-No comparison of changes in ACSCs admissions among areas with different impacts of SARS<br>-No correlation analysis between aggregate outpatient and inpatient statistics, because the health seeking behaviour might have varied among conditions with different severity and likelihood of acquiring SARS at health facilities
**Sasabuchi Y, et al [17]**	2017	Japan	Earthquake	N= 952 127 patients	To investigate the influence of the GEJE on long term health conditions in terms of ACSCs using a national inpatient database in Japan	-Admission for acute ACSCs significantly increased in the disaster area compared with other areas at 16-24 months after the Great East Japan Earthquake	-No measure of outpatient care or of the baseline health and socioeconomic status of the residents<br>-Validity of ACSC in Japanese clinical setting needs to be further evaluated<br>-Difference in admission policy between hospitals<br>-Unable to track whether patients admitted to other hospital after discharge<br>-"Out of pocket" payments for healthcare charges were exempted during the time of the study
**Runkle JR, et al [13]**	2013	US	Chlorine spill	All Medicaid patients admitted to hospitals/ED between 18 and 64	To estimate unmet PHC needs after a disaster	-Improved access to timely and effective primary care in the 24 months post-disaster<br>-PHC provided an alternate site of preventive and primary care in the 24 months after the disaster	-Small sample size<br>-ACSC rates not analyzed separately<br>-Data not generalizable
**Runkle JD, et al [14]**	2012	US	Chlorine spill	All Medicaid beneficiaries, representative of medically vulnerable individuals	To see whether ACSC rates among vulnerable subpopulations are sensitive to the impact of a disaster	-Significant increase in post—disaster hospital ACSC visits<br>-Significant decline in post-disaster ED discharges<br>-Elevated ACSC rates signal PHC issues that existed before the disaster<br>-Disaster exacerbates disparities in access<br>-Excess demand on the health system promotes discontinuity of care and disrupts access to care for vulnerable subgroups during recovery	-Fallacies in the design of the study (Secondary group not suitable)<br>-Possible confounding factors (health seeking behaviour, disease severity trends)
**Leuchter RK, et al. [12]**	2021	US	COVID-19	N= 904 patients without COVID-19 admitted in internal medicine at a tertiary hospital	To measure the number and change in potentially avoidable hospitalizations during COVID-19: 6 months in 2019 versus 6 months in 2020	-Reduced ACSC rates during COVID<br>-African-American were more hospitalized	-No incorporation of known medical and nonmedical contributors to health disparities (comorbidities)<br>-Assumption of a common population pool of patients between the 2 time periods<br>-Small sample size from a single health system<br>-No explanation of underlying causes of system inefficiencies<br>-Portion of pneumonia might be COVID-19+
**Rennert-May E. et al. [20]**	2021	Canada	COVID-19	Population of Alberta ED visits + hospital admission rates (diagnoses based on ICD-10)	To measure changes in number of hospital admissions and ED visits pre-and post-COVID-19; 7 months in 2019 versus 7 months in 2020	-Number of admissions significantly lower during COVID both for medical and surgical cases<br>-Number of ED visits for ACSCs decreased significantly post-COVID-19 public health measures<br>-Increased in-hospital mortality for medical admissions, decreased in-hospital mortality for surgical cases<br>-ED visits increase/decrease by number of COVID-19 cases, suggesting that population avoids ED when cases of COVID rise due to concern of transmission	-No data present of mortality outside of in-hospital rates<br>-Short time period<br>-No assessment of ambulatory care utilization
**Wright B. et al. [16]**	2021	US	COVID-19	Retrospective cross-sectional analysis of Medicaid beneficiaries between Jan 2018 and August 2020	To understand healthcare use and costs of individuals who were newly enrolled to Medicaid just prior to the pandemic versus those who were newly enrolled during the first Covid pandemic.	-New Medicaid enrollees during COVID: reductions in the probability of having an ED visit, a potentially avoidable hospitalization, a primary care visit, a dental visit, compared to new enrollees pre-COVID<br>-Elderly had more probability to experience a potentially avoidable hospitalization compared to younger people<br>-Women were less likely to have a potentially avoidable hospitalization, more likely to have an ED visit, and less likely to have a PHC visit<br>-Higher probability of health care use and costs among rural versus urban enrollees	-Only 6 months assessed<br>-Not generalizable
**Becker N. et al. [15]**	2022	US	COVID-19	Pre-pandemic: N=1.240.409 adults (>18) enrolled in a commercial health maintenance organisation<br>Pandemic: N=1.206.361 adults (>18) enrolled in a commercial health maintenance organisation	To describe trends in hospital admissions for ACSCs in the pre-pandemic period compared with the pandemic period.	-RR of having any ACSC hospitalisation in the pandemic period vs the pre-pandemic period was 0.72 (95% CI, 0.69-0.76; $P < .001$). This decrease was slightly larger in magnitude than the overall reduction in non-ACSC, non–COVID-19 hosp. rates.<br>-Large reductions were found in the RR of respiratory-related ACSC hospitalizations (aRR, 0.54; 95% CI, 0.50-0.58; $P < .001$), with non–statistically significant reductions in diabetes-related ACSCs (aRR, 0.91; 95% CI, 0.83-1.00; $P = .05$) and a statistically significant reduction in all other ACSC hospitalizations (aRR, 0.79; 95% CI, 0.74-0.85; $P < .001$)	-Sample included a commercially insured population of adults who were not necessarily representative of the entire US population<br>-Population under study is overall younger and healthier, as the overall ACSC hospitalisation rate is below the national average<br>-Analysis does not include important covariates, such as race and ethnicity, language spoken, income

File S3: Characteristics of the studies included
