# Supplementary material for: Using Ambulatory Care Sensitive Conditions to Assess Primary Health Care Performance during Disasters: A Systematic Review
Source: Int J Environ Res Public Health. 2022 Jul 27;19(15):9193. doi: 10.3390/ijerph19159193 (PMC9367847; doi:10.3390/ijerph19159193)
Supplement: Supplementary file 1 [file ijerph-19-09193-s001.zip › File S4.pdf]

	Chronic obstructive pulmonary disease/Asthma	Congestive heart failure	Arterial Hypertension	Diabtes mellitus	Angina	Dehydration and elektrolytes disturbances	Urinary Infections	Iron-deficiency anemia	Gastrointestinal bleeding	Nutritional deficiencies	Seizures	Sever Ear/Nose/Throat infections	Cellulitis	Pelvic Inflamm. Disease	Dental conditions	Gangrene	Influenza	Pneumonia
Leuchter et al., 2021	x	x	x	x														x
Runkle et al., 2013	x	x	x	x	x	x	x			x	x	x	x	x	x			x
Sasabuchi et al., 2017	x	x	x	x	x	x	x	x	x	x	x	x	x	x	x	x	x	x
Sasabuchi et al. 2018	x	x	x	x	x	x	x	x	x	x	x	x	x	x	x	x	x	x
Huang et al. 2009	x	x	x	x														
Runkle et al, 2012	x	x	x	x	x	x	x			x	x	x	x	x	x			x
Rennert—May 2021	x	x	x	x	x						x							x
Wright 2021	x	x	x	x	x		x				x							x
Becker 2022	x	x	x	x			x				x							x

File S4. ACSCs used in the reviewed articles
